# Proteinase-activated receptor 2 and disease biomarkers in cerebrospinal fluid in cases with autopsy-confirmed prion diseases and other neurodegenerative diseases

**DOI:** 10.1186/s12883-015-0300-x

**Published:** 2015-03-31

**Authors:** Zdenek Rohan, Magdalena Smetakova, Jaromir Kukal, Robert Rusina, Radoslav Matej

**Affiliations:** Department of Pathology and Molecular Medicine, National Reference Laboratory for Diagnostics of Human Prion Diseases, Thomayer Hospital, Prague, Czech Republic; Institute of Pathology, Third Medical Faculty of Charles University in Prague and Kralovske Vinohrady Teaching Hospital, Prague, Czech Republic; Department of Software Engineering, Faculty of Nuclear Science and Physical Engineering, Czech Technical University, Prague, Czech Republic; Thomayer Hospital, Department of Neurology, Prague, Czech Republic; Department of Neurology and Centre of Clinical Neuroscience, First Faculty of Medicine, Charles University in Prague, and General University Hospital in Prague, Prague, Czech Republic

**Keywords:** Proteinase-activated receptor 2, Tau, Phospho-tau, Beta-amyloid, Protein 14-3-3, Creutzfeldt-Jakob disease

## Abstract

**Background:**

Proteinase-activated receptor 2 (PAR-2) has been shown to promote both neurotoxic and neuroprotective effects. Similarly, other routinely used nonspecific markers of neuronal damage can be found in cerebrospinal fluid (CSF) and can be used as biomarkers for different neurodegenerative disorders.

**Methods:**

Using enzyme-linked immunosorbent assays and western blotting we assessed PAR-2, total-tau, phospho-tau, beta-amyloid levels, and protein 14-3-3 in the CSF of former patients who had undergone a neuropathological autopsy after death and who had been definitively diagnosed with a prion or other neurodegenerative disease.

**Results:**

We did not find any significant correlation between levels of PAR-2 and other biomarkers, nor did we find any differences in PAR-2 levels between prion diseases and other neurodegenerative conditions. However, we confirmed that very high total-tau levels were significantly associated with definitive prion diagnoses and exhibited greater sensitivity and specificity than protein 14-3-3, which is routinely used as a marker.

**Conclusions:**

Our study showed that PAR-2, in CSF, was not specifically altered in prion diseases compared to other neurodegenerative conditions. Our results also confirmed that very high total-tau protein CSF levels were significantly associated with a definitive Creutzfeldt-Jakob disease (CJD) diagnosis and should be routinely tested as a diagnostic marker. Observed individual variability in CSF biomarkers provide invaluable feedback from neuropathological examinations even in “clinically certain” cases.

**Electronic supplementary material:**

The online version of this article (doi:10.1186/s12883-015-0300-x) contains supplementary material, which is available to authorized users.

## Background

Protease-activated receptor 2 (PAR-2) belongs to the family of protease-activated receptors (PARs) that consists of four members: PAR-1, PAR-2, PAR-3, and PAR-4. PARs are activated by proteolytic cleavage of their extracellular N-terminus to unveil a neo-ligand that subsequently binds to the extracellular domain on the receptor itself and activates it irreversibly. PAR-1, PAR-3 and PAR-4 are mainly activated by thrombin, while PAR-2 is preferentially activated by trypsin. Intracellular PAR-activated signaling, via gene expression, influences cellular activity [1].

In the CNS, PARs are expressed on neurons, glia, ependymal cells and endothelial cells and modulate both neuroprotective and neurotoxic effects [2]. In a recent study using a murine prion disease model, PAR-2 knockout scrapie-inoculated mice showed delayed onset of symptoms and longer survival than PAR-2 wild-type mice [3]. This suggests a possible modulatory role for PAR-2 in the dynamics of prion diseases. Although, the complete role of PAR-2 in brain pathology remains unclear.

Cerebrospinal fluid (CSF) biomarkers are being increasingly used in the clinical diagnosis of neurodegenerative disorders. Total-tau (T-tau), phosphorylated tau (P-tau), beta-amyloid (Aβ) levels, and protein 14-3-3 status are routinely assessed in differential diagnostic workups of dementia. These biomarkers have also diagnostic potential for other neurodegenerative diseases such as Lewy body diseases [4,5] and frontotemporal lobar degenerations (FTLD) [6].

The aim of this study was to evaluate PAR-2 as a possible differential marker of neurodegenerative processes. As part of the study we also completed an *ante mortem* evaluation of T-tau, P-tau, Aβ levels, and 14-3-3 status and compared the results and associations with PAR-2 values.

## Methods

### Study design and case selection

The study was designed as a retrospective analysis of diagnostic samples collected by our institution. Patients referred to our institution for dementia (including possible/probable Creutzfeldt-Jakob disease; CJD) were clinically and neuropsychologically assessed, and then underwent neuroimaging and CSF examinations as part of a routine diagnostic workup.

Cases with a neuropathologically confirmed diagnosis of neurodegenerative disease and an *ante mortem* CSF analysis of T-tau, P-tau, Aβ, and protein 14-3-3 were included in the study. Of 59 patients, 36 had a neuropathologically confirmed prion disease and 23 were diagnosed with some other neurodegenerative disease (see Table 1 and the Additional file 1).

**Table 1:** **Summary of the obtained results for CJD and non-CJD cases**

**Cases**		**n**	**Male/Female**	**Disease duration [months]**	**Age at death [years]**	**PAR-2** ***[ng/ml]***	**T-tau** ***[pg/ml]***	**P-tau** ***[pg/ml]***	**Aβ** ***[pg/ml]***	**14-3-3**		
										**P**	**W**	**N**
CJD		36	12/24	6.8 ± 8.3 [1–48]	63.1 ± 8.3 [39–81]	8.78 ± 6.40 [1.25–28.49]	1111.47 ± 264.48 [195–1201]	52.69 ± 30.86 [16–166]	619.39 ± 297.34 [212–1407]	21	7	8
non-CJD		23	15/8	24.1 ± 22.2 [2–84]	71.0 ± 10.4 [53–90]	9.31 ± 8.71 [1.22–41]	632.43 ± 434.03 [65–1201]	47.48 ± 28.38 [15–103]	561.22 ± 421.20 [94–1672]	4	3	16
	AD	6	4/2	28.67 ± 29.87 [2–84]	70 ± 10.66 [54–82]	13.26 ± 14.62 [1.22–41]	926.83 ± 366.99 [268–1201]	53.83 ± 27.67 [29–102]	401 ± 409.04 [94–1109]	1	2	3
	FTLD-TDP	11	7/4	17.2 ± 14.86 [3–46]	70.8 ± 11.4 [53–90]	8.15 ± 5.96 [1.73–21.79]	558.27 ± 424.44 [134–1201]	39.10 ± 24.35 [15–94]	517.64 ± 241.35 [265–1065]	2	0	9
	VD	4	3/1	27.5 ± 31 [2–72]	70.5 ± 7.1 [62–79]	5.03 ± 1.61 [2.79–6.44]	547 ± 521.99 [65–1201]	52.25 ± 38.45 [20–103]	945 ± 755.50 [186–1672]	1	1	2
	PSP	2	1/1	38 ± 2.83 [36–40]	76.5 ± 17.68 [64–89]	12.36 ± 6.05 [8.08–16.64]	328 ± 239 [159–497]	65 ± 42.43 [35–95]	514 ± 55.15 [475–553]	0	0	2

Results are shown as *mean* ± *S.D. [min–max]*; *P* – positive, *W* – weakly positive, *N* – negative. AD – Alzheimer’s disease; FTLD-TDP – frontotemporal lobar degeneration with TDP-43 inclusions; PSP – progressive supranuclear palsy; VD – vascular dementia.

The patients or their relatives agreed with the storage of CSF samples and brain tissue for research purposes and signed informed consents. The study was approved by the Ethics committee of our institution, Thomayer Hospital, Prague.

### Cerebrospinal fluid analysis

Non-hemolytic CSF samples were briefly vortexed and T-tau, P-tau, Aβ, and PAR-2 levels were analyzed using commercially available ELISA kits (INNOGENICS® INNOTEST® hTAU Ag, cat. #80323, INNOTEST® PHOSPHO-TAU_(181P)_, cat. #80317, INNOTEST® β-AMYLOID_(1–42)_, cat. #80324 and USCN ELISA Kit for PAR-2, cat. #SEA852Hu). Protein 14-3-3 status was assessed using sodium-dodecyl sulphate polyacrylamide gel electrophoresis and western blot using polyclonal IgG antibody against protein 14-3-3 (K-19; Santa Cruz Biotechnology; sc-629), followed by chemiluminescent detection (Pierce® ECL Plus; #32132).

#### Neuropathology assessment

Brains were fixed in 10% formalin for 2 – 4 weeks. Samples were embedded in paraffin blocks and diagnosed using standardized recommendations [7]. A definite diagnosis of CJD was confirmed through neuropathological examination and western blot detection of the proteinase K resistant form of prion protein. In positive cases, the prion protein gene (*PRNP*) was analyzed for codon 129 polymorphisms and disease-associated mutations.

#### Statistical analysis

Patient data were split to CJD (n = 36) and non-CJD (n = 23) groups (Table 1). The Wilcoxon-Mann–Whitney (WMW) nonparametric test was used for the median equity hypothesis H0 at a significance level of 0.05 for six variables (age at death, duration of illness, and CSF levels of T-tau, P-tau, Aβ, and PAR-2). The Fisher exact test of independence hypothesis H0, in a 2 × 2 contingency table, was used for CJD/non-CJD group vs. categorical data at a significance level of 0.05 for two variables (protein 14-3-3, categorized T-tau). Significance levels were decreased using the Bonferroni correction for multiple testing. Due to the non-gaussian nature of experimental data, a non-parametric approach to correlation analyses was used. The Spearman rho correlation coefficient was calculated and the hypothesis of independence was tested using a critical level of 0.05.

## Results

Summarized results of case groups are included in Table 1, detailed results of each case are presented in the Additional file 1.

### PAR-2 CSF levels did not differ significantly among studied neurodegenerative diseases

Using the WMW and a Bonferroni correction of significance levels (0.05/6 = 0.0083), no statistically significant difference was found between PAR-2 CSF levels in the CJD and non-CJD groups (p-value = 0.085).

### Greatly increased T-tau levels, decreased disease duration and age at death as well as a positive protein 14-3-3 status were significantly associated with prion diseases

**Figure 1:**
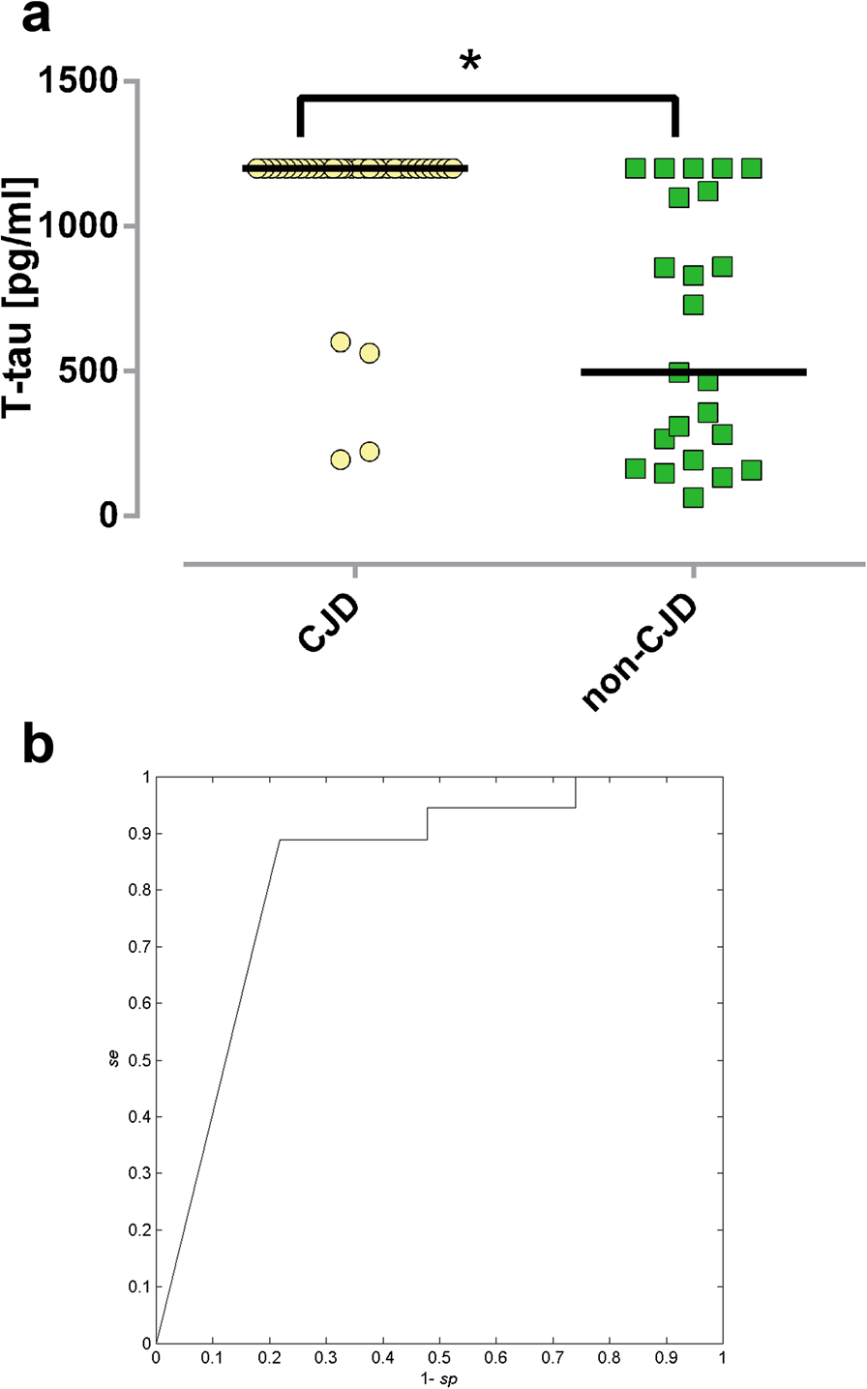
**Summary of T-tau results. a**. Box plot of individual T-tau values in CJD (n = 36) and non-CJD (n = 23) cases. Bars represent T-tau median values; * = p-value < 0.05. **b**. ROC diagram of T-tau values higher than 1200 pg/ml in CJD cases. *se* – sensitivity; *sp* – specificity.

Values of T-tau were significantly higher in the CJD group (p-value = < 0.001; Figure 1a), while disease duration (p-value < 0.001) and age of death (p-value = 0.006) were significantly lower in the CJD group when evaluated using the WMW test with a Bonferroni correction. No differences in P-tau and Aβ CSF values were found between evaluated groups.

Statistically significant dependences between groups and categorical variables were found for both groups (evaluated using the Fisher exact test with decreased significance levels and a Bonferroni correction of 0.05/2 = 0.025). Positive and weakly positive protein 14-3-3 status (p-value < 0.001, sensitivity 77.7%, specificity 69.5%) as well as T-tau values greater than 1200 pg/ml were shown to be significant indicators for a CJD diagnosis (p-value < 0.001, sensitivity 88.8%, specificity 78.2%). The ROC diagram of T-tau is presented in Figure 1b.

The mean levels of Aβ were lowest in AD compared to other neurodegenerations. However, the small number of AD cases did not allow confirmation of significance.

### No statistically significant correlation between PAR-2 and T-tau in CSF in either the CJD or the non-CJD group

We found no statistically significant correlation between PAR-2 and CSF levels of T-tau and protein 14-3-3 status in either the CJD group (T-tau R = 0.11, p = 0.51) or the non-CJD group (T-tau R = 0.01, p = 0.97). However, PAR-2 levels were significantly and inversely correlated with protein 14-3-3 status in the non-CJD group (R = −0.45; p = 0.03).

Other biomarkers, less relevant as prion disease biomarkers (e.g. P-tau and Aβ), were statistically significantly correlated with PAR-2 CSF levels (P-tau R = 0.4, p = 0.01; Aβ R = 0.4, p = 0.02).

## Discussion

Our goal was to evaluate PAR-2 CSF levels in patients with various neurodegenerative diseases. Our results showed that PAR-2 CSF levels did not differ between CJD and non-CJD groups, suggesting that changes in PAR-2 CSF levels are not specifically associated with prion disease. Based on our results, PAR-2 cannot serve as a biomarker for distinguishing prion diseases from other neurodegenerative diseases. However, based on recent results obtained in mice [3], PAR-2 may act as a nonspecific enhancer of neurodegenerative processes. This concept of PAR-2, as a neurodegeneration-promoting factor, has also been supported by other studies.

We also did not find any significant correlation between PAR-2 and T-tau CSF levels in the CJD or non-CJD groups. Interestingly, in the non-CJD group, protein 14-3-3 status correlated with PAR-2 CSF levels; however, this is most probably a biased observation since non-CJD cases with positive or weak protein 14-3-3 were also often associated with increased T-tau levels, which probably reflects neuronal damage rather than a relationship to PAR-2 mediated pathogenesis, although, it could also be involved in neuronal damage mechanisms in general.

Another goal of our study was to retrospectively evaluate results of CSF biomarkers routinely used in diagnostics of neurodegenerative diseases. Our results are in agreement with larger studies [8-13]. In our cohort, presence of protein 14-3-3 positivity and isolated T-tau CSF values were significantly higher in prion diseases than in other neurodegenerative diseases, regardless of whether the prion disease had a sporadic or genetic origin and regardless of codon 129 *PRNP* polymorphisms.

T-tau is generally considered to be a marker of neuronal damage in AD and lesions caused by ischemic brain injury, CNS infections, and epileptic seizures [14]. Human prion diseases are often characterized by sudden onset, rapid progression, extensive neuronal damage, and being rapidly fatal (months). T-tau levels may initially be low, but tend to increase during the disease course [15]. Isolated T-tau levels greater than 1200 pg/ml were almost always associated with prion disease. In non-prion neuropathology, we found high T-tau levels in one case of vascular dementia associated with severe ischemic white matter lesions (case 38), in three AD patients with rapid progression (cases 46–48), and in two cases of neuropathologically confirmed frontotemporal lobar degeneration with TDP-43 inclusions (FTLD-TDP; cases 54 and 59). In the AD subgroup (n = 6), T-tau values ranged from 497 pg/ml to over 1200 pg/ml, with the highest T-tau levels associated with either increased P-tau or decreased Aβ levels. Conversely, slowly progressive diseases, such as AD and FTLDs, generally have lower rates of neuronal damage and longer survival times (on the order of years). Additionally, they have rather low T-tau levels, although, levels can increase to over 1200 pg/ml during disease progression; a pattern similar to what was seen in this study.

Levels of P-tau and Aβ did not significantly differ between groups. Nevertheless, P-tau values in some CJD and non-AD-non-CJD cases reached the cutoff value that is usually regarded as specific for AD (median of 60 pg/ml) [16] and therefore could be a potential diagnostic pitfall in differential diagnostic workups for dementia. Moreover, in four of six cases of definite AD, we observed decreased (under 400 pg/ml) levels of Aβ in the CSF (cases 44–47). This result further supports the diagnostic utility of lower levels of Aβ in AD cases.

The strengths of our study were: (1) it only included neuropathologically confirmed cases with an *ante mortem* CSF analysis and (2) it excluded cases with mixed neuropathology. This approach provides objective results when studying prion disease and other neurodegeneration biomarkers.

## Conclusions

We showed that PAR-2 CSF levels do not differ among prion diseases and other neurodegenerative diseases. This may suggest a disease-nonspecific modulatory role in neurodegeneration processes. Our results also confirmed that very high T-tau CSF levels and 14-3-3 protein positivity are significantly associated with a definite CJD diagnosis. Individual variability in CSF biomarkers, however, was commonly observed, which led us to conclude that neuropathological examinations, of even “clinically certain” cases, can still provide invaluable feedback to clinicians.

## Additional file

Additional file 1:
**Cases included in the study.**
