# Supplementary material for: Proteinase-activated receptor 2 and disease biomarkers in cerebrospinal fluid in cases with autopsy-confirmed prion diseases and other neurodegenerative diseases
Source: BMC Neurol. 2015 Mar 31;15:50. doi: 10.1186/s12883-015-0300-x (PMC4392746; doi:10.1186/s12883-015-0300-x)
Supplement: Additional file 1: — Cases included in the study. [file 12883_2015_300_MOESM1_ESM.docx]

Additional file 1: Cases included in the study.

| case # | Age at death | Gender | Duration [months] | 14-3-3  status | T-tau  [pg/ml] | P-tau  [pg/ml] | Aβ  [pg/ml] | PAR-2  [ng/ml] | PRNP  codon 129 | Neuropathological diagnosis | Clinical diagnosis |
| --- | --- | --- | --- | --- | --- | --- | --- | --- | --- | --- | --- |
| 1 | 71 | F | 2 | N | 195 | 20 | 640 | 7,79 | MM | sCJD | RPD |
| 2 | 60 | F | 4 | P | 600 | 46 | 421 | 6,01 | MM | sCJD | CJD |
| 3 | 57 | F | 10 | N | 1201 | 114 | 1080 | 7,29 | MM | sCJD | CJD |
| 4 | 52 | M | 4 | P | 1201 | 62 | 476 | 23,7 | VV | sCJD | CJD |
| 5 | 54 | M | 3 | P | 1201 | 49 | 590 | 8,6 | MM | sCJD | CJD |
| 6 | 81 | F | 2 | P | 1201 | 39 | 446 | 28,49 | MM | sCJD | CJD |
| 7 | 62 | F | 1 | P | 1201 | 60 | 619 | 8,05 | MM | sCJD | CJD |
| 8 | 62 | F | 5 | P | 1201 | 72 | 497 | 17,23 | MV | sCJD | CJD |
| 9 | 68 | M | 6 | P | 1201 | 63 | 639 | 6,22 | VV | sCJD | CJD |
| 10 | 68 | M | 6 | P | 1201 | 42 | 351 | 6,75 | VV | sCJD | CJD |
| 11 | 68 | M | 4 | N | 223 | 44 | 255 | 4,26 | MM | sCJD | RPAD/CJD |
| 12 | 71 | F | 4 | P | 1201 | 166 | 1123 | 13,13 | VV | sCJD | CJD |
| 13 | 74 | F | 17 | P | 1201 | 21 | 481 | 8,8 | MV | sCJD | CJD |
| 14 | 49 | M | 3 | P | 1201 | 19 | 294 | 10,37 | MM | sCJD | CJD |
| 15 | 58 | M | 16 | W | 1201 | 66 | 1407 | 14,22 | MV | sCJD | CJD |
| 16 | 61 | F | 1 | P | 1201 | 41 | 812 | 7,02 | MM | sCJD | CJD |
| 17 | 59 | F | n/a* | N | 563 | 42 | 909 | 7,25 | MM | sCJD | CJD |
| 18 | 69 | M | 1 | P | 1201 | 50 | 1160 | 15,16 | MM | sCJD | CJD |
| 19 | 69 | F | 8 | P | 1201 | 49 | 680 | 12,86 | MM | sCJD | CJD |
| 20 | 65 | M | 2 | P | 1201 | 29 | 609 | 5,83 | MM | sCJD | CJD |
| 21 | 69 | F | 2 | P | 1201 | 102 | 1047 | 16,76 | MM | sCJD | CJD |
| 22 | 70 | F | 11 | P | 1201 | 48 | 927 | 8,30 | MV | sCJD | CJD |
| 23 | 53 | F | 4 | W | 1201 | 19 | 272 | 1,54 | MM | sCJD | CJD |
| 24 | 63 | F | 1 | N | 1201 | 18 | 231 | 1,42 | MM | sCJD | CJD |
| 25 | 71 | M | 2 | N | 1201 | 55 | 776 | 2,99 | MM | sCJD | CJD |
| 26 | 74 | F | 7 | P | 1201 | 58 | 329 | 1,58 | VV | sCJD | CJD |
| 27 | 62 | F | 12 | W | 1201 | 82 | 747 | 3,30 | MM | sCJD | CJD |
| 28 | 65 | F | 5 | W | 1201 | 37 | 600 | 2,85 | MM | sCJD | CJD |
| 29 | 71 | F | 1 | P | 1201 | 53 | 663 | 2,50 | MM | sCJD | CJD |
| 30 | 58 | F | 5 | W | 1201 | 21 | 229 | 1,25 | MM | sCJD | CJD |
| 31 | 58 | F | 8 | N | 1201 | 59 | 799 | 15,96 | MM | fCJD (E200K mutation) | CJD |
| 32 | 62 | F | 11 | W | 1201 | 20 | 518 | 5,42 | VV | fCJD (R208H mutation) | PSP/CJD |
| 33 | 53 | F | 11 | W | 1201 | 75 | 673 | 7,79 | MV | fCJD (E200K mutation) | CJD |
| 34 | 62 | F | 6 | P | 1201 | 48 | 315 | 1,77 | MM | fCJD (E200K mutation) | CJD |
| 35 | 39 | M | 48 | N | 1201 | 16 | 212 | 7,66 | MV | GSS (P102L mutation) | GSS |
| 36 | 65 | M | 4 | P | 1201 | 92 | 471 | 16,12 | MM | GSS (P102L mutation) | CJD |
| 37 | 62 | F | 12 | N | 65 | 25 | 410 | 2,79 | n/a | VaD | VaD/mtb |
| 38 | 69 | M | 2 | P | 1201 | 20 | 186 | 5,00 | n/a | VaD | VaD/mtb/CJD |
| 39 | 79 | M | 72 | N | 728 | 103 | 1512 | 5,92 | n/a | VaD | VaD/AD |
| 40 | 62 | F | 6 | W | 357 | 28 | 552 | 1,728 | n/a | VaD | CJD |
| 41 | 72 | M | 24 | N | 194 | 61 | 1672 | 6,44 | n/a | PSP | AD |
| 42 | 64 | M | 40 | N | 159 | 35 | 553 | 16,64 | n/a | PSP | PSP |
| 43 | 89 | F | 36 | N | 497 | 95 | 475 | 8,08 | n/a | AD (Braak stage V) | depression |
| 44 | 76 | M | 27 | W | 860 | 29 | 256 | 6,58 | n/a | AD (Braak stage V) | CJD |
| 45 | 68 | M | 84 | N | 830 | 102 | 94 | 10,62 | n/a | AD (Braak stage VI) | AD |
| 46 | 78 | F | 2 | N | 1201 | 65 | 143 | 40 | n/a | AD (Braak stage VI) | CJD |
| 47 | 62 | M | 36 | P | 1201 | 58 | 125 | 1,215 | n/a | AD (Braak stage VI) | AD/FTD |
| 48 | 82 | F | 7 | W | 1201 | 35 | 679 | 3,659 | n/a | AD (Braak stage IV) | CJD |
| 49 | 54 | M | 16 | N | 268 | 34 | 1109 | 16,47 | n/a | FTLD-TDP | FTD |
| 50 | 62 | M | 4 | P | 1121 | 54 | 595 | 13,35 | n/a | FTLD-TDP | CJD |
| 51 | 90 | M | 46 | N | 857 | 69 | 1065 | 8,38 | n/a | FTLD-TDP | AD |
| 52 | 75 | M | 9 | N | 148 | 26 | 265 | 5,22 | n/a | FTLD-TDP | MSA/CJD |
| 53 | 70 | M | n/a | N | 164 | 34 | 748 | 10,68 | n/a | FTLD-TDP | FTD |
| 54 | 80 | F | 12 | N | 1099 | 94 | 281 | 11,68 | n/a | FTLD-TDP | FTD/AD |
| 55 | 69 | M | 12 | N | 282 | 40 | 494 | 4,71 | n/a | FTLD-TDP | FTD |
| 56 | 78 | M | 3 | N | 467 | 16 | 339 | 6,72 | n/a | FTLD-TDP | CJD |
| 57 | 58 | M | 20 | N | 311 | 33 | 647 | 21,79 | n/a | FTLD-TDP | FTD/MSA/CJD |
| 58 | 53 | F | 20 | N | 134 | 15 | 348 | 2,321 | n/a | FTLD-TDP | CJD |
| 59 | 82 | F | 40 | P | 1201 | 21 | 360 | 3,111 | n/a | FTLD-TDP | CJD |

Supplemental table legend: codon 129 polymorphisms: **MM** – methionine/methionine, **MV** – methionine/valine, **VV** – valine/valine; protein 14-3-3 status: **P** – positive, **W** – weak, **N** – negative; **AD** – Alzheimer’s disease; **FTD** – frontotemporal dementia; **FTLD-TDP** – frontotemporal lobar degeneration with phosphorylated TDP-43 inclusions without motor neuron involvement; **GSS** – Gerstmann-Sträussler-Scheinker disease; **MSA** – multiple system atrophy; **mtb** – metabolic encephalopathy; **fCJD** – familial Creutzfeldt-Jakob disease; **PSP** – progressive supranuclear palsy**; RPAD** – rapidly progressive Alzheimer’s disease; **RPD** – rapidly progressive dementia; **sCJD** – sporadic Creutzfeldt-Jakob disease; **VaD** – vascular dementia; **n/a** – not available; **n/a*** – diagnosis made on brain biopsy
